# Resolving inflammation: The impact of antiretroviral therapy on macrophage traffic in and out of the CNS

**DOI:** 10.1371/journal.ppat.1013180

**Published:** 2025-12-01

**Authors:** Zoey K. Wallis, Cecily C. Midkiff, Miaoyun Zhao, Patrick Autissier, Soon Ok Kim, Addison Q. Amadeck, Yiwei Wang, Maia Jakubowski, Tricia H. Burdo, Andrew D. Miller, Qingsheng Li, Xavier Alvarez, Robert V. Blair, Kenneth C. Williams

**Affiliations:** 1 Morrissey College of Arts and Sciences, Biology Department, Boston College, Chestnut Hill, Massachusetts, United States of America; 2 Tulane National Biomedical Research Center, Covington, Louisiana, United States of America; 3 Nebraska Center for Virology, School of Biological Sciences, University of Nebraska-Lincoln, Lincoln, Nebraska, United States of America; 4 Department of Medicine, Rutgers Institute for Translational Medicine and Science, Robert Wood Johnson School of Medicine, New Brunswick, New Jersey, United States of America; 5 Department of Population Medicine and Diagnostic Sciences, Section of Anatomic Pathology, College of Veterinary Medicine, Cornell University, Ithaca, New York, United States of America; 6 Imaging PET-CT and OIL Core, Southwest National Primate Research Center, San Antonio, Texas, United States of America; University of Wisconsin, UNITED STATES OF AMERICA

## Abstract

The effects of antiretroviral therapy (ART) and treatment interruption on myeloid cell egress from the central nervous system (CNS) during human immunodeficiency virus (HIV) and simian immunodeficiency virus (SIV) infection remain poorly defined. We hypothesized that CNS macrophages normally traffic out of the CNS, accumulate during viral infection and inflammation, and are released as inflammation resolves. To test this, we administered intracisternal (i.c.) injections of two different colored fluorescent superparamagnetic iron oxide nanoparticles (SPION) to SIV-infected macaques either early in infection (12–14 dpi) or 30 days before necropsy. SPION are preferentially taken up by perivascular, meningeal, and choroid plexus macrophages, enabling us to track macrophage turnover, infection, and migration. In non-infected macaques, SPION^+^ macrophages trafficked from the CNS to peripheral sites including the deep cervical lymph node (dCLN), lumbar lymph nodes, spleen, and dorsal root ganglia (DRG). With SIV infection, these cells accumulated in the CNS and showed reduced peripheral trafficking. ART decreased the number of SPION+ perivascular macrophages, and to a lesser extent, meningeal or choroid plexus macrophages. After ART interruption, SPION^+^ perivascular and choroid plexus macrophage numbers remained stable, whereas SPION^+^ meningeal macrophages increased. ART eliminated SIV-RNA^+^ perivascular macrophages, leaving few- scattered SIV-RNA^+^ cells in the meninges and choroid plexus. Following ART interruption, perivascular macrophages remained virus-negative, but scattered viral RNA^+^ meningeal macrophages persisted. In non-infected macaques, SPION^+^ macrophages trafficked to the dCLN, spleen, and DRG, but this trafficking diminished with SIV infection and AIDS with SIVE. Importantly, SIV-RNA^+^ SPION^+^ macrophages that exited the CNS were cleared by ART and did not reappear after treatment interruption. Using two differently colored SPION to assess establishment of CNS viral reservoirs, we observed higher numbers of early-labeled macrophages within and outside the CNS in animals with AIDS and SIVE, ART treatment, and ART interruption. These findings support a model in which SIV-infected perivascular macrophages seed an early CNS viral reservoir, while the meninges and choroid plexus undergo continual viral seeding during infection. ART reduces trafficking of infected macrophages out of the CNS and clears the perivascular macrophage reservoir, but SIV-RNA+ meningeal macrophages can persist, in low numbers, and can rebound after ART interruption.

## Author summary

We used nanoparticles injected into CNS ventricles-cerebrospinal fluid of SIV-infected macaques, which are taken up by brain macrophage populations, to examine their accumulation during SIV infection and CNS inflammation, as well as their behavior during resolution of infection. We found that macrophages accumulate in the CNS with SIV infection, and that with antiretroviral therapy (ART) and reduced CNS inflammation, brain macrophages—contrary to prior assumptions—can exit the CNS. We discuss these findings in the context of whether brain macrophages might carry virus out of the CNS during HIV infection.

## Introduction

Antiretroviral therapy (ART) has reduced the incidence of severe human immunodeficiency virus (HIV)-associated neurocognitive disorders (HAND) yet the prevalence of mild and asymptomatic HAND and HIV-associated neuropathy remains and has in fact increased [1]. While durable ART diminishes HIV and simian immunodeficiency virus (SIV) infection, latently infected myeloid cells persist in the CNS and may be a source of viral recrudescence where macrophage traffic out of the CNS could result in viral redistribution [2–8]. Distinct CNS macrophages, some of which originate from bone marrow (BM) monocytes, can be HIV- and SIV-infected and establish the CNS viral reservoir [9–11]. These include perivascular macrophages, meningeal macrophages, and macrophages in the choroid plexus. CNS perivascular macrophages monitor the interface between cerebrospinal fluid (CSF) and blood [12–15], are HIV and SIV-RNA^+^ and DNA^+^ as early as 3–7 days post-infection [16,17], and make up the CNS viral reservoir [6,9,10,16]. Resident meningeal macrophages, derived from mesodermal precursors in the yolk sac, are repopulated from BM at a slow, consistent rate [13,18–21]. The choroid plexus contains BM-derived stromal and Kolmer macrophages that turnover continuously [22,23] and are HIV- and SIV-RNA^+^ and DNA^+^ [24–28]. The resident CNS macrophage—microglia—have a minimal capacity for self-renewal and may be a viral reservoir, less so than perivascular macrophages [13,29,30]. In normal and HIV-infected humans and SIV-infected non-human primates CNS macrophage inflammation occurs that contributes to turnover and an accumulation of BM-derived perivascular, and meningeal and CP macrophages [31–34]. Less well-studied and more pressing is the effect of ART and ART interruption on these CNS macrophages with HIV and SIV infection with regards to not only their accumulation but their potential to traffic out of the CNS.

Minimally, there are three entry routes of BM and blood-derived monocytes/macrophage into the CNS including (i) migration directly from the blood via postcapillary venules in the parenchyma (perivascular macrophage), (ii) migration from postcapillary venules in the meninges (meningeal macrophage), and (iii) migration to and through the choroid plexus (stromal and Kolmer macrophage) into the CSF and subarachnoid space (SAS) (meningeal and perivascular macrophage) [35–37]. Using CD34^+^ autologous hematopoietic stem cells transplanted in rhesus macaques, we found perivascular macrophages are continuously renewed from BM, similar to what others have shown to occur in humans and rodents [12,14,15,38–42]. We and others have shown early traffic (3–7 days) of virally infected monocytes/macrophages into the CNS seeds the brain with HIV and SIV [43–45] and blocking subsequent traffic with the anti-VLA4-α4β1 antibody (natalizumab) alone blocks CNS virus and resolves neuronal injury [43–45]. While previous efforts have focused on blocking the migration of bone marrow-derived monocytes/macrophages into the CNS, whether macrophages can exit the brain and traffic to the periphery under normal conditions and SIV infection is not well studied, but has been recently demonstrated by us [46]. That CNS macrophage populations are reservoirs of HIV and SIV is well established however, their ability to reseed the periphery with CNS-derived virus has been suggested but not determined.

Potential pathways for exit of immune cells and fluid leaving the CNS are topics of current interest but the dynamics of viral infection and ART, and the resolution of inflammation are understudied. Historically, this is due in part to the belief in a lack of CNS-draining lymphatics [35]. Using intracisternal and intraparenchymal injection (i.c.) of dyes and fluorescently labeled antigen-presenting cells (APC), traffic of APC and drainage of CSF to the deep cervical lymph nodes (dCLN) is shown to occur normally at a rate that is increased with inflammation [19,47–52]. Ablation of meningeal lymphatics and the dCLN reduces such traffic and effectively reduces clinical symptoms in experimental autoimmune encephalomyelitis (EAE) [19,47–52]. Another potential pathway follows CSF from the fourth ventricle into the central canal and the lateral and median apertures to the SAS along the entirety of the spinal cord [53,54]. This path also allows for exchange between the CSF in the central canal, CSF enveloping the spinal cord in the subarachnoid space (SAS) via perivascular spaces, which allows for the traffic of fluid and cells out of the CNS [53,54]. While eloquent, studies using experimental injection or transplantation of labeled cells and fluid into the CNS parenchyma can result in CNS injury and/or disruption of the blood-brain barrier (BBB) and CNS parenchymal immune cell activation [48,49]. Additionally, these studies have largely been done in rodents. To date, it is unknown whether macrophages leave the CNS in humans or non-human primates normally and with HIV or SIV infection. Similarly, the effects of ART on CNS macrophage viral reservoirs, CNS inflammation and macrophage traffic out, viral recrudescence, and CNS viral redistribution to the periphery are not known.

In this study we use i.c. injection of fluorescently labeled superparamagnetic iron oxide nanoparticles (SPION) to study perivascular, meningeal, and choroid plexus macrophage accumulation, traffic, and infection. We previously showed SPION preferentially labeled these macrophages in non-infected macaques in the CNS that can migrate out to secondary lymphoid organs including the dCLN, spleen, and DRG, and they accumulate in the CNS with SIV infection [55]. In the current study, we analyzed CNS macrophage accumulation and traffic out in SIV-infected animals with acquired immunodeficiency syndrome (AIDS) and SIV-induced encephalitis (SIVE), animals on ART, and 4 weeks post-ART interruption. We hypothesized that CNS inflammation with SIV infection and AIDS results in accumulation of SPION^+^ macrophages and ART promotes the resolution of CNS facilitating SPION^+^ macrophage egress. We find retention of SPION^+^ macrophages in the CNS of animals with AIDS and SIVE and decreased macrophage traffic out. ART reduces SPION^+^ macrophage numbers in the CNS and increases their numbers in the periphery. ART clears SIV-RNA^+^SPION^+^ perivascular macrophages but not SIV-RNA^+^SPION^+^ meningeal and choroid plexus macrophage. There is a rebound of plasma virus, SIV-RNA^+^SPION^+^ meningeal macrophages, but not CNS perivascular macrophages with ART interruption. SPION^+^ SIV-RNA^+^ macrophages are found in the periphery (dCLN, spleen, and DRG) that are cleared with ART. These findings are discussed in the context of CNS macrophage inflammation, infection-accumulation and a viral rebound with ART interruption.

## Results

### ART reduces SPION^+^ perivascular macrophages but not meningeal or choroid plexus macrophages

**Fig 1:**
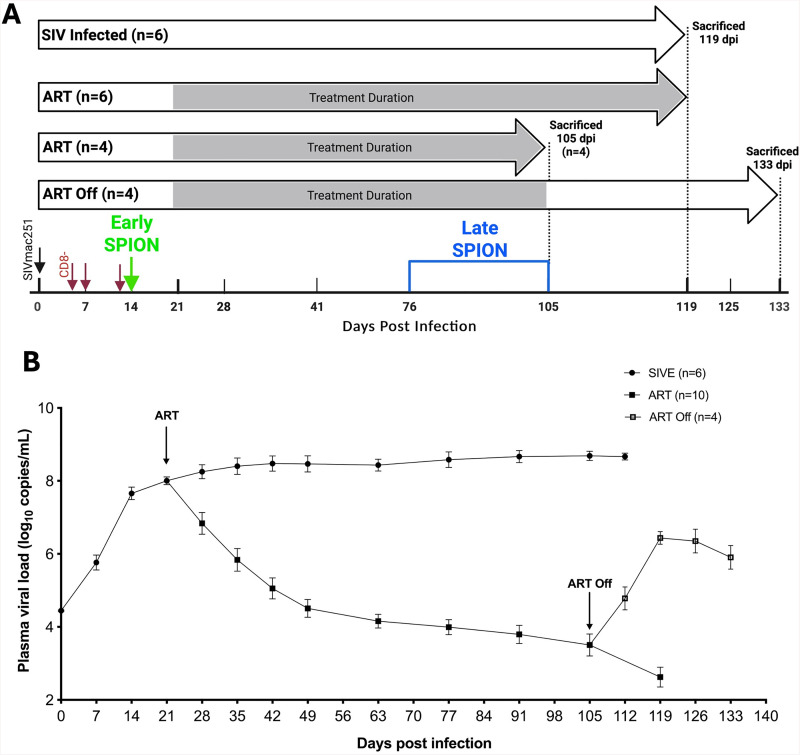
Study design and plasma viral load. (A) Twenty rhesus macaques were infected with SIVmac251 (black arrow) and CD8 T lymphocyte depleted on 5, 7, and 12 dpi (red arrows). Fourteen macaques received ART once daily starting 21 dpi until 105 – 119 dpi. A set of animals was sacrificed on ART (n = 10), or four weeks following ART interruption (n = 4). N = 6 animals that did not receive ART were sacrificed when they developed AIDS 83-126 dpi. All ART animals were used for CNS tissues studies and a subset of N = 4 were used for studies of peripheral tissues as the remaining animals did not have these tissues available. Macaques received i.c. SPION injection early (12-14 dpi, Green Early SPION) and late (30 days prior to sacrifice, Red Late (pseudo-colored blue) SPION). The study design figure was created using BioRender with appropriate licensure. (B) Plasma viral load was measured longitudinally in all animals. Prism was used for graphing and statistical analysis using Kruskal-Wallis nonparametric test followed by Dunn’s post-hoc test, with significance accepted at p < 0.05.

**Fig 2:**
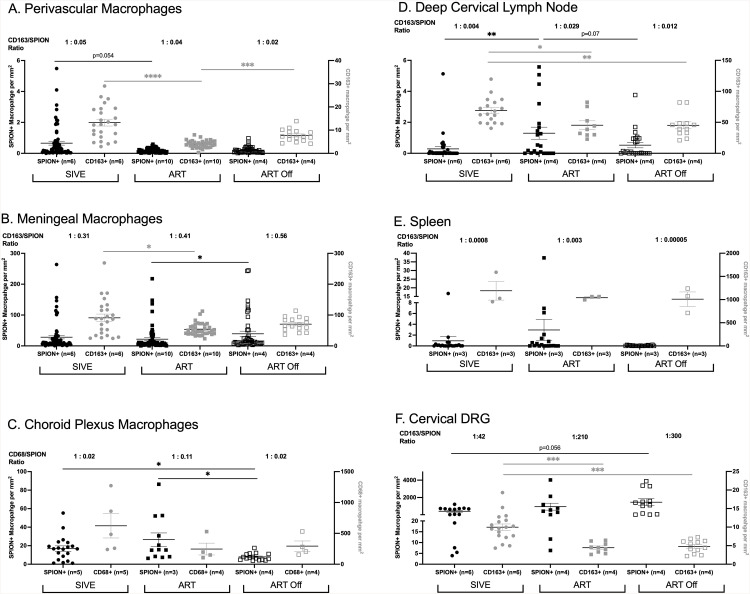
ART reduces the number of SPION+ perivascular macrophages and increases the number of SPION+ macrophages trafficking out. Single-label immunohistochemistry for CD163 with Prussian Blue staining was used with light microscopy to count numbers of SPION^+^ macrophages (black shapes) and macrophage without SPION (grey shapes) in non-ART SIV-infected animals with encephalitis (SIVE, circles), SIV-infected macaques sacrificed on ART (ART, closed squares)(SIVnoE), and SIV-infected macaques 4 weeks post ART interruption (ART Off, open squares). Comparisons are made between the (A) perivascular space, (B) meninges, (C) choroid plexus, (D) dCLN, (E) spleen, and (F) DRG. Each data point represents cell counts from entire tissue sections and is expressed as the number of positive cells per mm^2^. Above each data set are the average ratio of the total CD163 or CD68 macrophages over the number of SPION+ macrophages. Prism was used for graphing and statistical analysis Kruskal-Wallis nonparametric test was followed by Dunn’s post-hoc test, *P < 0.05, **P < 0.01, and ****P < 0.0001.

We analyzed 3 animal cohorts: SIV-infected that developed acquired immunodeficiency syndrome (AIDS) and SIV-induced encephalitis (SIVE) (n = 6), SIV infected animals on ART that did not develop AIDS or SIVE (SIVnoE)(n = 10), and SIV-infected animals studied 4 weeks after ART interruption (n = 4) (Table 1 and Fig 1). Overall, there are fewer numbers of SPION^+^ perivascular macrophages than SPION^+^ meningeal macrophages (range, 50-195x) and SPION^+^ choroid plexus macrophages (range, 1-5x) (Fig 2A-C and S1 Table). Because there are greater total numbers of macrophages in the meninges and choroid plexus compartments than the perivascular macrophage compartment, and thus more cells that can take up SPION, we determined the ratio of total CD163^+^ and CD68^+^ macrophages to CD163^+^ and CD68^+^ SPION^+^ macrophages in each compartment to normalize the data (perivascular SIVE 1:0.05; ART 1:0.04; ART Off 1:0.02)(meningeal SIVE 1:0.31; ART 1:0.41; ART Off 1:0.56)(choroid plexus SIVE 1:0.02; ART 1:0.11; ART Off 1:0.02) (Fig 2). Importantly, the ratio of total macrophages to SPION^+^ macrophages did not change within compartments between treatment groups. Animals with AIDS and SIVE have 0.66± 0.13 SPION^+^/mm^2^ SPION^+^ perivascular macrophages that were reduced to 0.18±0.02 SPION^+^/mm^2^ with ART and did not change with ART interruption (0.21± 0.20 SPION^+^/mm^2^) (p = 0.054) (Fig 2A). SIVE animals had 28±5.8 SPION^+^/mm^2^ SPION^+^ meningeal macrophages and no decrease with ART (22±4.9 SPION^+^/mm^2^) and significantly increased numbers with ART interruption compared to ART (ART Off: 39±8.6 SPION^+^/mm^2^) (p < 0.05) (Fig 2B). There are 18±13 SPION^+^/mm^2^ SPION^+^ macrophages in the choroid plexus of SIVE animals that trend toward an increase with ART (26±25 SPION^+^/mm^2^) and significantly decreased with ART interruption (8.0±3.7 SPION^+^/mm^2^) (p < 0.05) (Fig 2C). The decrease in the number of SPION^+^ macrophages in the choroid plexus following ART interruption may correspond with the increased numbers of SPION^+^ macrophages in the meninges as a result of macrophage traffic into the CNS. Overall, these data are consistent with the accumulation of perivascular macrophages with SIVE that is reduced with ART and an accumulation of SPION^+^ meningeal and choroid plexus macrophages that are unaffected by ART.

**Table 1:** Animals Used in Study.

Cohort	Animal ID	Treatment	DPI at Death	CNS Pathology	Days with Green SPION	Days with Red SPION
SIVE (n = 6)	IK28	Untreated	100	SIVE	86	3
	JE87	Untreated	119	SIVE	105	14
	JD29	Untreated	126	SIVE	112	7
	KN69	Untreated	83	SIVE	69	6
	KT79	Untreated	119	SIVE	105	22
	LB12	Untreated	115	SIVE	101	10
ART (n = 4)	JJ86	ART	105	SIVnoE	89	26
	KD67	ART	105	SIVnoE	89	26
	KM38	ART	105	SIVnoE	90	27
	LK25	ART	105	SIVnoE	93	28
ART (n = 6)	JH68	ART	118	SIVnoE	108	28
	JI15	ART	118	SIVnoE	108	28
	GI68	ART	119	SIVnoE	109	29
	JB07	ART	119	SIVnoE	109	29
	GH18	ART	120	SIVnoE	110	30
	JF58	ART	120	SIVnoE	110	30
ART Off (n = 4)	KD12	ART Interruption	133	SIVnoE	119	28
	JV76	ART Interruption	132	SIVnoE	118	27
	JV78	ART Interruption	133	SIVnoE	119	28
	KE98	ART Interruption	133	SIVnoE	119	28

Twenty SIV-infected, CD8^+^ T-lymphocyte-depleted rhesus macaques were used in this study. Sections of cortical CNS tissue were examined blinded on sample identity by a veterinary pathologist. Animals are diagnosed based on histopathology as having SIV encephalitis (SIVE) with productive CNS viral replication, the presence of multinucleated giant cells (MNGC), and macrophage accumulation, or SIV infection with no encephalitis (SIVnoE). All macaques received i.c. SPION injections early (12–14 dpi, Green Early SPION) and late (26–30 days before sacrifice, Red Late (pseudo colored-blue) SPION). N = 14 animals began ART 1x daily beginning at 21 dpi and N = 4 animals had ART interrupted (ART Off) for 4 weeks prior to sacrifice. DPI = days post infection.

#### ART decreases total perivascular, meningeal, and choroid plexus macrophage numbers.

We next assessed the effects of ART and ART interruption on the accumulation of total macrophages in the perivascular space, meninges, and choroid plexus compartments. SIVE animals have 13±1.5 CD163^+^/mm^2^ CD163^+^ perivascular macrophages that significantly decrease with ART (4±0.26 CD163^+^/mm^2^, p < 0.0001) and significantly increase with ART interruption (7.7±0.66 CD163^+^/mm^2,^ p < 0.001) (Fig 2A). Similarly, ART significantly decreases the number of meningeal and choroid plexus macrophages (meninges SIVE: 91±12 CD163^+^/mm^2^; ART: 54±3.3 CD163^+^/mm^2^) (choroid plexus SIVE: 622±441 CD68^+^/mm^2^; ART: 246±192 CD68^+^/mm^2^) (p < 0.0001) (Fig 2B-C). Only CD68^+^ macrophages were counted in the choroid plexus because of a limitation of tissue availability and CD68 macrophages are the primary macrophage population in this compartment. Following ART interruption, there is a 1.5-fold increase in the number of meningeal macrophages compared to ART that did not reach statistical significance and no increase in the number of choroid plexus macrophages (meninges ART: 54±3.3 CD163^+^/mm^2^; ART Off: 70±5.5 CD163^+^/mm^2^) (choroid plexus ART: 246±192 CD68^+^/mm^2^; ART Off: 293±167 CD68^+^/mm^2^) (Fig 2B-C). This suggests there is an increased accumulation of perivascular, meningeal, and choroid plexus macrophages with SIVE that is reduced with ART, does not significantly rebound in the meninges and choroid plexus, but results in increased perivascular macrophages 4 weeks post ART interruption.

### There is increased traffic of SPION^+^ macrophages out of the CNS with ART

In recent work, following i.c. SPION inoculation of non-infected and SIV-infected animals, we found SPION^+^ macrophage in the optic nerve, nasal septum, cribriform plate, borders at the interface of the CNS and the periphery, and in the dCLN, spleen, and dorsal root ganglia (DRG) [46]. In contrast to the CNS and consistent with our previous findings, there are fewer SPION^+^ macrophages in the periphery of animals with SIVE than animals on ART (dCLN SIVE: 0.28±0.15 SPION^+^/mm^2^; ART: 1.3±0.39 SPION^+^/mm^2^) (spleen SIVE: 0.95±3.5 SPION^+^/mm^2^; ART: 3.0±8.6 SPION^+^/mm^2^) (DRG SIVE: 420±104 SPION^+^/mm^2^; ART: 947±400 SPION^+^/mm^2^) (Fig 2D-F). There is a trend of decreased numbers of SPION^+^ macrophages in the periphery following ART interruption compared to ART (dCLN ART Off: 0.53±0.18 SPION^+^/mm^2^) (spleen ART Off: 0.051±0.07 SPION^+^/mm^2^) (Fig 2D and 2E). In contrast, the number of SPION^+^ macrophages in the DRG have a non-significant trend toward increase with ART (2.3-fold) and ART interruption (3.5-fold) relative to animals with SIVE (DRG ART: 947±400 SPION^+^/mm^2^; ART Off: 1458±398 SPION^+^/mm^2^) (Fig 2F). Although the number of SPION+ macrophages in the DRG did not significantly increase with ART interruption, there is a reduced ratio of total CD163^+^ macrophages to CD163^+^SPION^+^ macrophages in the DRG (SIVE 1:42, ART 1:213, ART Off 1:304), suggestive of increased SPION^+^ macrophage leaving the CNS via the DRG with ART interruption. Importantly, we also found scattered SPION^+^ macrophage within lumbar lymph nodes (S1 Fig). Overall, these data are consistent with SPION^+^ macrophages that are retained in the CNS with SIV-infection and CNS inflammation with increased CNS SPION^+^ macrophages trafficking out to the periphery with ART, and resolution of CNS inflammation.

#### ART decreases the number of total macrophages in the periphery.

In parallel with studies of SPION^+^ macrophages in the periphery, we assessed the number of macrophages in the same tissue with ART and ART interruption. In the dCLN, SIVE animals have 69±4.8 CD163^+^/mm^2^ CD163^+^ macrophages that significantly decrease with ART (45±7.0 CD163^+^/mm^2^, p < 0.05) that are similar after ART interruption (ART Off 45±5.6 CD163^+^/mm^2^) (Fig 2D). Similarly, there are decreased numbers of macrophages in the spleen and significantly decrease macrophage numbers in the DRG of ART animals compared to SIVE (Spleen SIVE: 1,190±348 CD163^+^/mm^2^; ART: 1,041±40 CD163^+^/mm^2^) (DRG SIVE: 10±0.89 CD163^+^/mm^2^; ART: 4.5±0.45 CD163^+^/mm^2^) (p < 0.005) (Fig 2E and 2F). There are no differences in the number of macrophages in the spleen and DRG between the ART and ART interruption cohorts (Spleen ART: 1,041±40 CD163^+^/mm^2^; ART Off 1,008±270 CD163^+^/mm^2^) (DRG ART: 4.5±0.45 CD163^+^/mm^2^; ART Off 4.9±0.51 CD163^+^/mm^2^) (Fig 2E and 2F). These data demonstrate an increased accumulation of macrophages in the dCLN, spleen, and DRG with SIV infection and AIDS that is reduced with ART and does not rebound with ART interruption.

### ART reduces SPION^+^ and total SIV-RNA^+^ macrophages and gp41^+^ cells in the CNS and periphery

**Fig 3:**
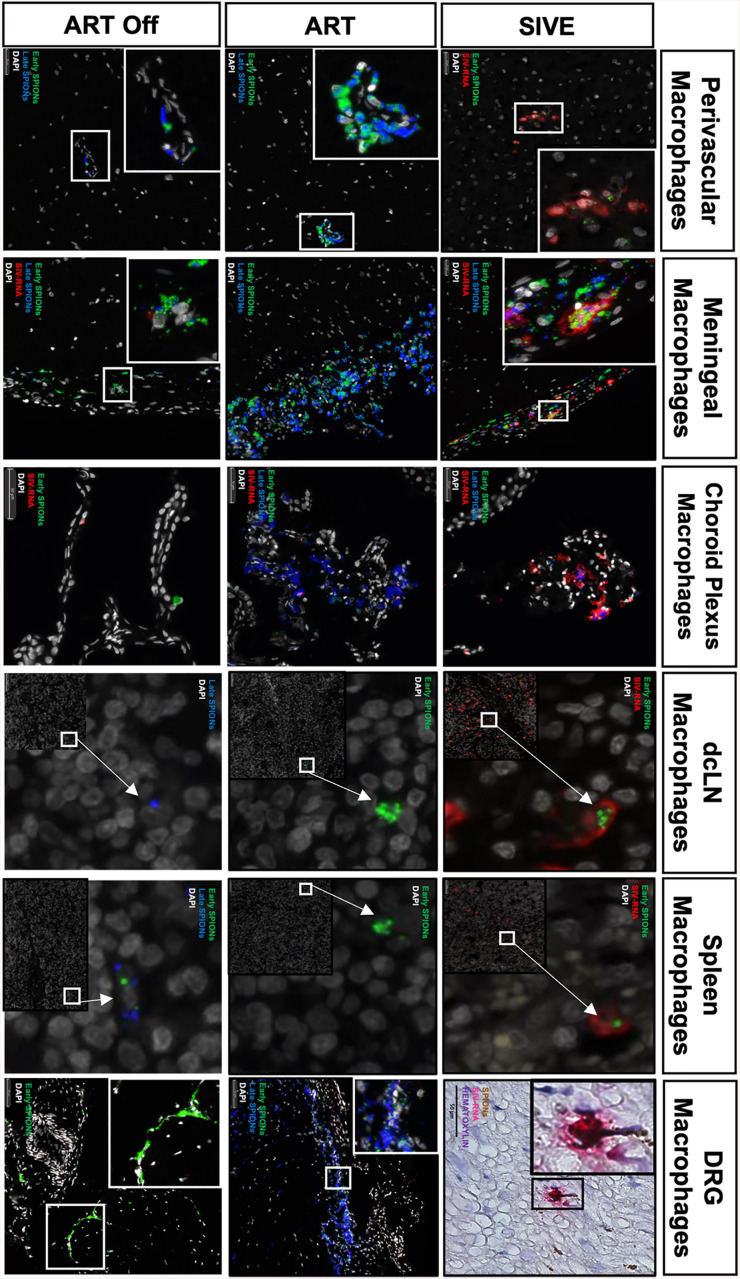
SPION^+^ SIV-RNA^+^ macrophages in the parenchyma, meninges, choroid plexus, dCLN, spleen, and DRG of animals with AIDS and SIVE. SIV-RNA (red) was detected in the CNS perivascular space, meninges, choroid plexus, and outside of the CNS in the dCLN, spleen, and DRG using RNAscope in situ hybridization. Early (green) SPION were i.c. injected 12-14 dpi and Late (pseudo-colored colored blue) SPION were i.c. injected 30 days prior to sacrifice. Images were captured from scanned tissue sections. Nuclei (gray) are stained with DAPI.

**Fig 4:**
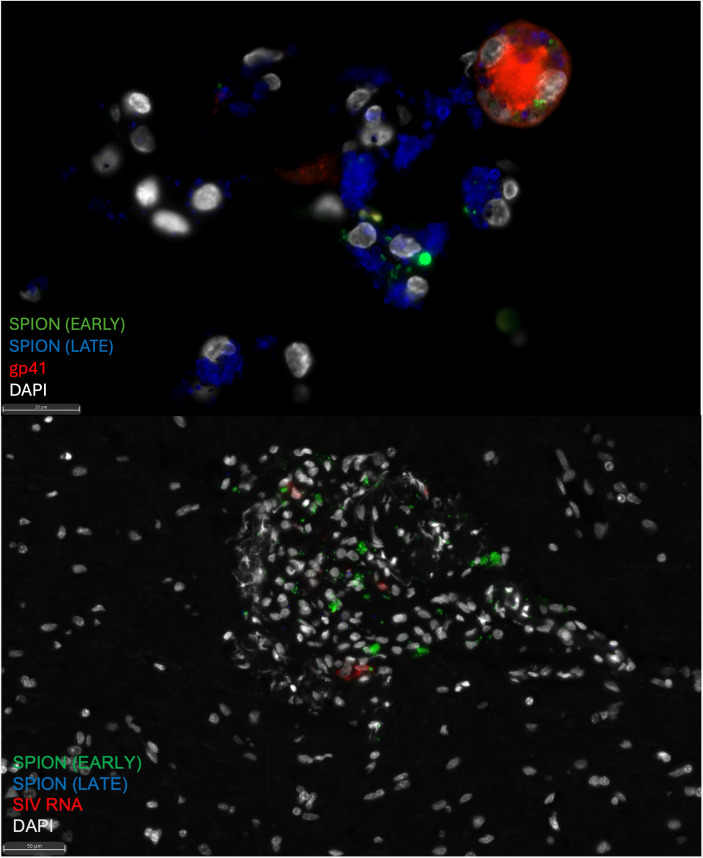
GP41^+^ SPION^+^ MNGC in the CNS of an animal with AIDS and SIVE. (A) SPION (Green (early) and Blue (late)) localize to a gp41^+^ (red) MNGC in the cerebellum. (B) SPION (Green (early) and Blue (late)) localize to a SIV-RNA^+^ (red) macrophages within SIVE lesions in the CNS cortical tissue. Nuclei (grey) are stained with DAPI.Outside the CNS, there are significantly increased numbers of SPION^+^ SIV-RNA^+^ and SIV-RNA^+^ macrophages in AIDS and SIVE animals that are significantly reduced with ART and do not rebound with ART interruption (p < 0.05) (Table 2). There are few scattered CD163^+^ and CD68^+^ SIV-RNA^+^ macrophages in the dCLN, spleen, and DRG in SIVE animals that are eliminated with ART and did not rebound following ART interruption. (Table 2). In addition, AIDS and SIVE animals have significantly more SPION^+^ SIV-DNA^+^ and SIV-DNA^+^ cells in the dCLN (2.67 to 133.50 vDNA^+^ cells/mm^2^ range), spleen (0 to 8.01 vDNA^+^ cells/mm^2^ range), and DRG (0 to 0.03 vDNA^+^ cells/mm^2^ range) that are not detected with ART and do not rebound with ART interruption (p < 0.05) (Table 2). Overall, the data are consistent with ART reducing the number of productively infected and SPION^+^ virally infected macrophages in the CNS and periphery, where productive SIV-RNA^+^-gp41^+^ numbers rebound in the meninges, choroid plexus, dCLN, and spleen but not in CNS perivascular macrophages with ART interruption.

Using SIV-RNAscope and immunohistochemistry for SIV-RNA^+^ and SIV-gp41^+^ cells, we counted the number of SPION^+^ SIV-productively infected cells in the CNS and assessed the effects of ART and ART interruption (Table 2 and Fig 3). Overall, AIDS and SIVE animals have numbers of SIV-RNA^+^, SIV-DNA^+^, and gp41^+^ macrophages with and without SPION in the CNS and periphery that are significantly reduced with ART (Table 2 and Fig 3). Within the CNS, there are greater numbers of SIV-RNA^+^SPION^+^ meningeal (25x) and choroid plexus macrophages (3.2x) versus perivascular macrophages SIVE animals (Table 2 and Fig 3). There are significantly increased numbers of vRNA^+^ and gp41^+^ macrophages, including multi-nucleated giant cells (MNGC), localized primarily in CNS lesions—some of which contain SPION^+^ (Table 2 and Fig 4). In SIVE animals, SIV-DNA^+^ cells are primarily in lesions within the parenchyma (0 to 0.95 vDNA^+^ cells/mm^2^ range) and a few scattered cells in the meninges (0 to 0.15 vDNA^+^ cells/mm^2^ range) (Table 2). With ART, there is a significant reduction of SIV-RNA^+^ and gp41^+^ perivascular macrophages (CD163^+^ p < 0.005; CD68^+^ p < 0.0001), macrophages in the meninges (CD163^+^ p < 0.005; CD68^+^ p < 0.005), but not the choroid plexus (CD68^+^ p = 0.17) (Table 2). ART fully eliminates SIV-RNA^+^SPION^+^ perivascular macrophages while few scattered SIV-RNA^+^SPION^+^ meningeal (1 SPION^+^SIV-RNA^+^ macrophage) and choroid plexus macrophages (10 SPION^+^SIV-RNA^+^ macrophages) persist (Fig 3 and Table 2). With ART interruption, there are few scattered SPION^+^SIV-RNA^+^ macrophages and gp41^+^ cells in the meninges, but no SPION^+^SIV-RNA^+^ or SIV-RNA^+^ perivascular macrophages or macrophages in the choroid plexus (Table 2). Staining for GP41 and SIV-RNA^+^CD163^+^ double label macrophage in the choroid plexus was not performed due issues with tissue fixation that do not allow for IF microscopy.

**Table 2:** SPION^+^ SIV-RNA^+^ macrophages and gp41^+^ cells are reduced with ART and rebound in the meninges following ART interruption.

Tissue	Cohort	SIV-RNA^+^ Macrophage			SIV-DNA^+^ Cells		gp41^ +^ Cells^+^	
		CD163^+^ vRNA^+^	CD68^+^ vRNA^+^	SPION^+^	vDNA^+^	SPION^+^		
**A.**								
**Parenchyma**	SIVE	4.8 ± 1.9	9.5 ± 6.4	0.018 ± 0.033	0.24 ± 0.36 *	0.0012 ± 0.002	0.32 ± 0.52	
		[4,784]	[4,0524,]	[38]	[721]	[3]	[7,146]	
		334 mm²	452 mm²	1,885 mm²	2,948 mm²	2,948 mm²	3,217 mm²	
	ART	0.01 ± 0.01 **	0 ± 0 *	0 ± 0 *	0.0027 ± 0.0043	0 ± 0	0.02 ± 0.01 ***	
		[10]	[0]	[0]	[6]	[0]	[27]	
		396 mm²	539 mm²	1308 mm²	2,337 mm²	2,337 mm²	1,810 mm²	
	ART Off	0 ± 0 ***	0.01 ± 0.01	0 ± 0 *	0 ± 0	0 ± 0	0.02 ± 0.02 ****	
		[0]	[3]	[0]	[0]	[0]	[63]	
		199 mm²	892 mm²	1,843 mm²	2,431 mm²	2,431 mm²	2,761 mm²	
**Meninges**	SIVE	71 ± 48	38.3 ± 13.4	12.0 ± 25.8	0.03 ± 0.06	0.0093 ± 0.022	11.8 ± 9.5	
		[2,683]	[313]	[965]	[2]	[1]	[1474]	
		13 mm²	7.5 mm²	103 mm²	93 mm²	93 mm²	122 mm²	
	ART	0.13 ± 0.14 **	0 ± 0 *	0.004 ± 0.011 **	0 ± 0	0 ± 0	0 ± 0 ****	
		[8]	[0]	[1]	[0]	[0]	[0]	
		19 mm²	20 mm²	78 mm²	94 mm²	94 mm²	68 mm²	
	ART Off	0 ± 0**	0 ± 0 *	0.08 ± 0.13 *	0 ± 0	0 ± 0	0.15 ± 0.24 ****	
		[0]	[0]	[4]	[0]	[0]	[12]	
		9 mm²	17 mm²	71 mm²	96 mm²	96 mm²	78 mm²	
**Choroid Plexus**	SIVE	NA	7.1 ± 6.5	3.0 ± 4.6	NA	NA	NA	
			[55]	[120]				
			10 mm²	31 mm²				
	ART	NA	0 ± 0	0.40 ± 0.33	NA	NA	NA	
			[0]	[10]				
			14 mm²	71 mm²				
	ART Off	NA	0 ± 0	0.04 ± 0.08	NA	NA	NA	
			[0]	[1]				
			39 mm²	49 mm²				
**B.**								
**Tissue**	**Cohort**	**SIV-RNA**^**+**^ **Cells**	**SIV-RNA**^**+**^ **Macrophage**			**SIV-DNA+ Cells**		**gp41**^**+**^ **Cells**
			**CD163**^**+**^ **vRNA**^**+**^	**CD68**^**+**^ **vRNA**^**+**^	**SPION** ^**+**^	**vDNA** ^**+**^	**SPION** ^**+**^	
**dCLN**	SIVE	962 ± 819	157 ± 47	48.7 ± 22.5	1.0 ± 1.7	55.6 ± 68.8 *	0 ± 0	70.0 ± 74.0
		[165,745]	[352]	[146]	[70]	[125]	[0]	[20382]
		343 mm²	0.75 mm²	3 mm²	172 mm²	2.25 mm²	2.25 mm²	226 mm²
	ART	0.80 ± 0.66 **** †	3.3 ± 5.3 ****	0 **	0	0 ± 0	0 ± 0	0.14 ± 0.24 *
		[116]	[22]	[0]	[0]	[0]	[0]	[22]
		307 mm²	2.5 mm²	2.5 mm²	185 mm²	2.25 mm²	2.25 mm²	127 mm²
**Spleen**	SIVE	1.04 ± 1.5	97 ± 38	19.4 ± 28.6	0.008 ± 0.02	3.1 ± 4.3	0 ± 0	76.0 ± 68.0
		[665]	[218]	[43]	[8]	[7]	[0]	[2745]
		1717 mm²	0.75 mm²	2 mm²	610 mm²	2.25 mm²	2.25 mm²	36 mm²
	ART	0.31 ± 0.33 **	0 **	0	0	0 ± 0	0 ± 0	0.72 ± 0.90 *
		[17]	[0]	[0]	[0]	[0]	[0]	[13]
		1611 mm²	1.5 mm²	4.5 mm²	738 mm²	2.25 mm²	2.25 mm²	18 mm²
	ART Off	2.3 ± 1.5	8.5 ± 12.4 **	0.45 ± 0.77	0	0.45 ± 0.77	0 ± 0	16 ± 14
		[44]	[19]	[1]	[0]	[1]	[0]	[394]
		2168 mm²	0.75 mm²	2 mm²	516 mm²	2.25 mm²	2.25 mm²	24 mm²
**DRG**	SIVE	214 ± 53	1.6 ± 1.7	0.23 ± 0.11	0.03 ± 0.08	0.2 ± 0.2	0 ± 0	1.0 ± 1.0
		[112,333]	[259]	[17]	[157]	[3]	[0]	[364]
		128 mm²	36 mm²	73 mm²	110 mm²	121 mm²	121 mm²	303 mm²
	ART	0.1 ± 0.04 *	0	0	0 *	0 ± 0	0 ± 0	0 *
		[37]	[0]	[0]	[0]	[0]	[0]	[0]
		87 mm²	99 mm²	64 mm²	79 mm²	147 mm²	147 mm²	147 mm²
	ART Off	14 ± 21 *	0	0	0 *	0 ± 0	0 ± 0	0 *
		[5273]	[0]	[0]	[0]	[0]	[0]	[0]
		122 mm²	48 mm²	58 mm²	107 mm²	183 mm²	183 mm²	242 mm²

SIV-RNA^+^ macrophages and cells are detected using ultrasensitive SIV-RNAscope with double-labeled CD163^+^ and CD68^+^ and GP41 using IHC. Whole tissue sections were analyzed and the number of positive cells assessed using indirect immune-fluorescence (IF) microscopy. A minimum of 2 CNS cortical regions and average of 3 peripheral tissue sections were analyzed per animal. Counts are the mean ± SEM of the number of viral RNA^+^ cells, CD163^+^ and CD68^+^ macrophages, SPION-labeled viral RNA^+^ macrophages, and gp41^+^ cells. [N=] is total number of cells counted. Prism was used for graphing and statistical analysis Kruskal-Wallis nonparametric test was followed by Dunn’s post-hoc test where asterisks indicate significant differences from SIVE cohort, *P < 0.05, **P < 0.01, and ****P < 0.0001. † Indicates significant differences compared to ART animals (P < 0.05).

In parallel to the above studies, we assessed cell-associated SIV-RNA and DNA in FACsorted CD14^+^ monocytes (98% purity), CD3 + T Cells (99% purity), and plasma virus longitudinally (S2 Fig). CD14^+^ monocytes and CD3 + T Cells were sorted as previously described [56,57]. Overall, with ART, plasma virus load decreases 4.5 logs to approximately 1x10^2^_log10_copies/ml (Fig 1b). Plasma viral load does not decrease to undetectable levels because of the short duration of ART, and they are persistently CD8 lymphocyte-depleted. At 21 days p.i. there is an average of ~6.9 x 10^4^ SIV-vRNA and ~7.6 x 10^1^ SIV-vDNA copies per 10^6^ monocytes (n = 8). At the same time point, with ART, cell-associated SIV-RNA and DNA in CD14^+^ monocytes decreased from ~2.5 x 10^5^ SIV-vRNA and ~1.9 x 10^3^ SIV-vDNA copies per 10^6^ monocytes to 4.0 x 10^4^ SIV-vRNA and 7.5 x 10^3^ SIV-vDNA copies per 10^6^ monocytes (n = 4). At necropsy, non-ART animals with SIVE had ~ 4.4 x 10^5^ SIV-vRNA and ~9.7 x 10^3^ SIV-vDNA copies per 10^6^ monocytes, and ART animals have undetectable monocyte SIV-RNA and -DNA (n = 4). Four weeks following ART interruption, at necropsy, monocyte associated SIV-RNA and DNA rebounded to ~5.2 x 10^3^ SIV-vRNA and 3.7 x 10^1^ SIV-vDNA copies per 10^6^ monocytes (n = 4).

### ART reduces early SPION^+^ perivascular macrophages in the CNS that corresponds to an increase in early SPION^+^ macrophages in the periphery

**Fig 5:**
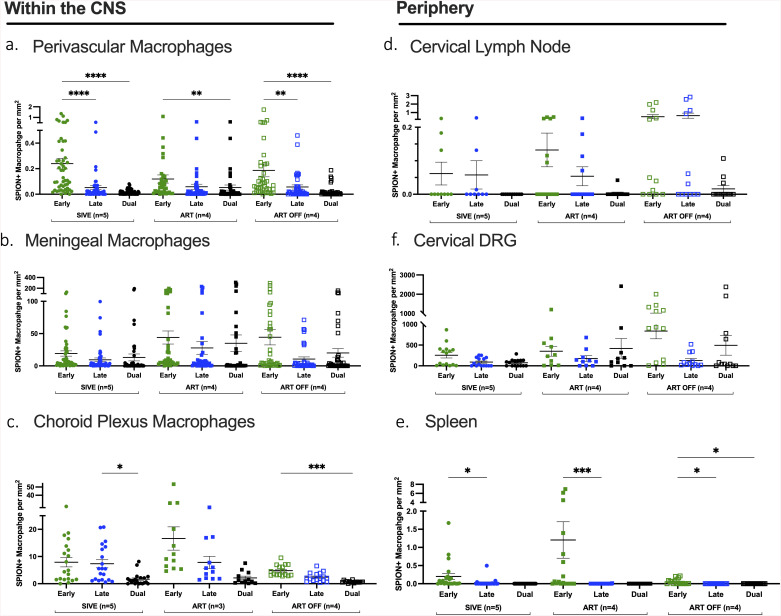
SPION-containing macrophages in and outside of the CNS are primarily labeled with SPION from early injection. Distribution of early, late, and dual-labeled SPION-labeled macrophage within the CNS (A) parenchyma, (B) meninges, (C) choroid plexus, and in the periphery including (D) dCLN, (E) spleen, and (F) DRG. Data points are the number of SPION positive cells detected per tissue section per animal with a minimum of 2 tissue sections analyzed per animal. Prism was used for graphing and statistical analysis Kruskal-Wallis nonparametric test was followed by Dunn’s post-hoc test, *P < 0.05, **P < 0.01, and ****P < 0.0001.

To better define the timing and establishment of CNS macrophage viral reservoirs and the effect of ART and ART interruption on CNS macrophage retention and traffic out, we injected two different colored SPION. Green, fluorescent SPION were injected i.c. 12–14 days post-infection (Early), and red fluorescent SPION (pseudo-colored blue) were injected 30 days prior to sacrifice (between 3–28 days prior to sacrifice, (Late)) (Table 1 and Fig 1). Our preliminary studies and work by others demonstrate SPION are internalized by macrophages within 30 minutes and remain intact in animals for greater than 120 days (the duration of our experiments) [58,59]. We analyzed SPION-labeled cells that have early, late, or both (dual-labeled with both early and late) SPION. There are significantly greater numbers of early versus late and dual-SPION^+^ perivascular macrophages in AIDS and SIVE animals, ART, and ART interruption animals (p < 0.0001) (Fig 5A and Tables 3 and S2). In contrast, there are equivalent numbers of early, late, and dual-labeled meningeal macrophages in SIVE, ART, and ART interruption animals (Fig 5A and 5B and Table 3). The distribution of early and late SPION^+^ choroid plexus macrophages is similar to the meninges with equivalent numbers of early, late, and dual SPION^+^ macrophages. There are slightly greater numbers of early (SIVE: 3-fold, ART 4-fold, ART Off 4.6-fold) versus late or dual-labeled choroid plexus macrophages (Table 3). Overall, these data suggest the CNS perivascular macrophage viral reservoir is established early in infection and meningeal and choroid plexus macrophages have ongoing recruitment of macrophages that increases with the development of AIDS and SIVE.

**Table 3:** Distribution of early, late, and dual SPION^+^ macrophage in the CNS and periphery.

Tissue	Cohort	Early SPION^+^ Macrophage	Late SPION^+^ Macrophage	Dual SPION^+^ Macrophage
A.				
**Parenchyma**	SIVE	0.24 ± 0.04	0.05 ± 0.02	0.02 ± 0.003
	ART	0.12 ± 0.03 *	0.06 ± 0.02	0.05 ± 0.02
	ART Off	0.19 ± 0.05	0.06 ± 0.02	0.02 ± 0.01
**Meninges**	SIVE	19 ± 4.0	9.5 ± 2.6	13 ± 5.4
	ART	44 ± 10	28 ± 9.8	35 ± 5.4
	ART Off	44 ± 12	11 ± 3.3	20 ± 6.7
**Choroid Plexus**	SIVE	7.9 ± 1.7	7.3 ± 1.5	1.6 ± 0.5
	ART	17 ± 4.3	7.8 ± 2.3	2.1 ± 0.70
	ART Off	4.8 ± 0.51	2.4 ± 0.42	0.67 ± 0.09
B.				
**dCLN**	SIVE	0.06 ± 0.03	0.06 ± 0.04	0 ± 0
	ART	0.13 ± 0.05	0.05 ± 0.03	0.003 ± 0.003
	ART Off	0.51 ± 0.24	0.64 ± 0.30 †	0.02 ± 0.01
**Spleen**	SIVE	0.20 ± 0.08	0.03 ± 0.02	0 ± 0
	ART	1.2 ± 3.0 ***	0.0007 ± 0.0007	0 ± 0
	ART Off	0.05 ± 0.02 †††	0 ± 0	0 ± 0
**DRG**	SIVE	225 ± 67	90 ± 27	352 ± 22
	ART	352 ± 120	177 ± 68	419 ± 238
	ART Off	836 ± 186	128 ± 50	494 ± 237

Whole tissue sections were analyzed, and the number of positive cells were identified using indirect-fluorescence. A minimum of 2 CNS cortical tissues were analyzed, and an average of 3 peripheral sections were examined per animal, per tissue region. Counts are the mean ± SEM of the number of SPION^+^ macrophages (Early, Late, or Dual (both early and late) SPION). SIVE, SIV encephalitis; dCLN, deep cervical lymph node; DRG, dorsal root ganglia. Prism was used for graphing and statistical analysis Kruskal-Wallis nonparametric test was followed by Dunn’s post-hoc test where asterisks indicate significant differences from SIVE cohort, *P < 0.05, **P < 0.01, and ****P < 0.0001. † Indicates significant differences from ART cohort, †P < 0.05, and †††P < 0.001.

### ART animals have greater numbers of early SPION^+^ macrophages in the periphery compared to AIDS animals with SIVE

Outside the CNS there is a trend of greater numbers of early than late or dual SPION^+^ macrophages in the in the dCLN, spleen, and DRG with SIVE, ART, and ART interruption although these did not reach statistical significance (Fig 5C and Table 3). With ART, there are greater numbers of early SPION^+^ macrophages in the spleen and few-to-no late or dual SPION^+^ macrophages (Fig 5D and Table 3). In the DRG, there are greater numbers of early and dual SPION^+^ macrophages compared to late in animals with AIDS and SIVE, ART, and following ART interruption (Fig 5E and Table 3). We next analyzed the number of early, late, and dual SPION^+^ virally infected macrophages in the CNS and periphery. The SPION^+^SIV-RNA^+^ perivascular macrophages in SIVE animals are primarily labeled with early SPION, that are eliminated with ART, and do not rebound with ART interruption (Table 4). In contrast there are greater numbers of Dual SPION^+^-SIV-RNA^+^ meningeal macrophages than early or late SPION in SIVE animals (Table 4) choroid plexus SPION^+^ SIV-RNA^+^ macrophages primarily have late SPION that are not eliminated with ART (Table 4). In the dCLN, SIV-RNA^+^ SPION^+^ macrophages in the dCLN are primarily late inoculated that are eliminated with ART and do not rebound with ART interruption (Table 4). In the spleen of SIVE animals, there are equivalent numbers of early and late injected SPION^+^SIV-RNA^+^ macrophages that are eliminated with ART and do not rebound following ART interruption (Table 4). These data support the early establishment of the CNS macrophage viral reservoir, ART resolving CNS inflammation with subsequent traffic of macrophages out early, and increased traffic of virally infected macrophages out of the CNS that occurs late during infection with AIDS and SIVE.

**Table 4:** ART eliminates SPION^+^SIV-RNA CNS perivascular macrophages and SPION^+^SIV-RNA^+^ macrophages outside the CNS but SPION^+^SIV-RNA^+^ meningeal and choroid plexus macrophages persist.

Tissue	Cohort	Early SPION^+^ SIV-RNA^+^ Macrophage	Late SPION^+^ SIV-RNA^+^ Macrophage	Dual SPION^+^ SIV-RNA^+^ Macrophage
A.				
**Parenchyma**	SIVE	0.012 ± 0.027	0.005 ± 0.008	0.002 ± 0.003
		[22]	[13]	[3]
	ART	0.0 ± 0.0	0.0 ± 0.0	0.0 ± 0.0
		[0]	[0]	[0]
	ART Off	0.0 ± 0.0	0.0 ± 0.0	0.0 ± 0.0
		[0]	[0]	[0]
**Meninges**	SIVE	2.1 ± 3.0	3.0 ± 6.0	7.0 ± 17
		[191]	[250]	[524]
	ART	0.0 ± 0.0	0.004 ± 0.01	0.0 ± 0.0
		[0]	[1]	[0]
	ART Off	0.1 ± 0.04	0.01 ± 0.04	0.03 ± 0.07
		[1]	[1]	[2]
**Choroid Plexus**	SIVE	0.61 ± 0.62	1.9 ± 3.5	0.50 ± 0.68
		[23]	[77]	[20]
	ART	0.0 ± 0.0	0.29 ± 0.52	0.18 ± 0.27
		[0]	[0]	[3]
	ART Off	0.0 ± 0.0	0.0 ± 0.0	0.042 ± 0.084
		[0]	[0]	[1]
B.				
**dCLN**	SIVE	0.07 ± 0.14	0.9 ± 1.7	0.0 ± 0.0
		[20]	[50]	[0]
	ART	0.0 ± 0.0	0.0 ± 0.0	0.0 ± 0.0
		[0]	[0]	[0]
	ART Off	0.0 ± 0.0	0.0 ± 0.0	0.0 ± 0.0
		[0]	[0]	[0]
**Spleen**	SIVE	0.0025 ± 0.0	0.004 ± 0.009	0.0 ± 0.0
		[3]	[5]	[0]
	ART	0.0 ± 0.0	0.0 ± 0.0	0.0 ± 0.0
		[0]	[0]	[0]
	ART Off	0.0 ± 0.0	0.0 ± 0.0	0.0 ± 0.0
		[0]	[0]	[0]
**DRG**	SIVE	NA	NA	0.03 ± 0.08 •
				[157]
	ART	NA	NA	0.0 ± 0.0 •
				[0]
	ART Off	NA	NA	0.0012 ± 0.008 •
				[3]

SIVE, SIV encephalitis; dCLN, deep cervical lymph node; DRG, dorsal root ganglia. Counts are the mean ± SEM of the number of viral RNA^+^ cells and SPION-labeled viral RNA^+^ macrophages. Whole tissue sections were analyzed to obtain the number of positive cells using indirect fluorescence microscopy. A minimum of n = 2 cortical CNS tissues and an average of n = 3 peripheral sections were examined per animal, per tissue region. • Indicates early and late SPION was not assessed due to tissue fixative and inability to use IF markers.

## Discussion

### Macrophage accumulation and distribution in the CNS during AIDS and The impact of ART

Few studies have investigated CNS macrophage accumulation and egress under non-infected conditions and in the context of HIV/SIV infection, antiretroviral therapy (ART), and ART interruption. Using i.c. injection of SPION, which label perivascular, meningeal, and choroid plexus macrophages, we found that animals with AIDS and SIVE have increased numbers of CNS SPION⁺ macrophages, consistent with macrophage accumulation within the CNS. There was a differential compartmental distribution of SPION⁺ macrophages, with meningeal macrophages outnumbering perivascular macrophages by 50- to 200-fold, and meningeal macrophages outnumbering choroid plexus macrophages by 1- to 5-fold. These differences are consistent with the normally greater numbers of meningeal and choroid plexus macrophages relative to perivascular macrophages. Notably, the ratio of total macrophages to SPION⁺ macrophages within each compartment did not differ among SIVE, ART-treated, and ART-interrupted animals.

ART reduced the number of SPION⁺ perivascular macrophages, such that AIDS-SIVE animals had approximately four times more SPION⁺ perivascular macrophages than ART-treated animals. In contrast, ART did not alter SPION⁺ macrophage numbers in the meninges or choroid plexus. Following ART interruption, we observed increased SPION⁺ meningeal macrophages and decreased SPION⁺ choroid plexus macrophages relative to ART-treated animals. Increased meningeal SPION⁺ macrophages after ART interruption may reflect enhanced trafficking of bone marrow–derived macrophages from the choroid plexus to the CSF, resulting in secondary accumulation within the meninges.

We note that SPION^+^ macrophages in the perivascular space, dCLN, and the spleen typically contain fewer SPION per cell than those in the meninges and DRG. These differences likely reflect the distinct fluid-SPION interfaces in each anatomical compartment [18,60,61]. The intrinsic turnover rate of macrophages—both under baseline conditions and after SPION uptake, as well as SIV infection and ART—likely also contributes. It is plausible that once a macrophage is heavily loaded with SPION, certain egress pathways, such as the cribriform route, become less efficient. Thus, SPION uptake may slow macrophage exit from the CNS and contribute to compartment-specific macrophage accumulation, particularly in the meninges. Conversely, macrophages acquiring fewer SPION may retain greater migratory capacity.

Our data and previous observations by us and others suggest that SPION, unlike dextran-based dyes injected i.c., do not passively leak out of the CNS [33,48,62–64]. This is supported by the particle size used and by the absence of non–cell-associated SPION inside or outside the CNS. Although SPION degradation and subsequent exit from the CNS has been reported [65,66], this is unlikely within our < 1-year timeframe of our studies, and is due to the stability of the polymer coating used [64,67–69]. Clearance through the kidneys or induction of iron-related programmed cell death (via ROS generation and lipid peroxidation) are more plausible fates [64,67–70]. Overall, these data support differential turnover among perivascular, meningeal, and choroid plexus macrophages and highlight ART-induced reductions primarily in perivascular macrophages. That macrophages can exit tissues and traffic to draining lymph nodes has been demonstrated in rodent models [48,49,71] and likely occurs in the human CNS, although this has not been directly studied in primates to date.

### Differential turnover rates across CNS macrophage compartments

CNS macrophages undergo a basal turnover rate that increases with inflammation induced in our study by SIV infection [38]. Less well understood is how ART influences such turnover and how macrophage accumulation evolves across the course of viral infection. By injecting two differently colored SPION early and late, we observed greater numbers of early-labeled than late- or dual-labeled macrophages across all tissues and treatment conditions.

Perivascular macrophages were predominantly labeled by early SPION, suggesting a resident, low-turnover population whose turnover increases with SIV infection [33,72,73]. This is consistent with earlier reports of early recruitment of CD163 ⁺ bone marrow–derived perivascular macrophages into the CNS during SIV infection [12,38,41,72,73]. Greater early SPION labeling may also reflect limited SPION uptake capacity within this populationIn contrast, meningeal macrophages exhibited equivalent numbers of early- and late-labeled SPION⁺ cells across SIVE, ART-treated, and ART-interruption animals, consistent with a more stable, locally maintained population that is not substantially affected by ART status. The fluid dynamics of the meninges and DRG, which remain in continuous contact with CSF containing SPION, may promote the even distribution of early, late, and dual-labeled macrophages in these compartments.

Choroid plexus macrophages also contained equivalent early and late labeling but low dual labeling across all groups, consistent with high monocyte/macrophage turnover and traffic. Ongoing macrophage turnover and egress to the periphery may contribute to CNS lesion resolution and prevent sustained inflammation, aligning with recent rodent studies demonstrating macrophage exit from parenchymal tissues to draining lymph nodes [74,75].

Across peripheral tissues—including the dCLN, spleen, and DRG—we observed more early-labeled SPION⁺ macrophages, suggesting delayed clearance of labeled cells from the CNS rather than rapid drainage of free SPION. The predominance of early-labeled cells may also reflect the egress of a dendritic cell–like myeloid population from the perivascular space, as suggested by rodent studies [48,49]. We did not directly quantify the exit of specific CNS macrophage subpopulations or identify their precise routes of egress; such studies will be important for understanding CNS inflammation and its resolution, particularly in diseases such as multiple sclerosis and HIV infection during ART.

### Macrophage egress from the CNS to peripheral tissues

Several pathways for macrophage egress from the CNS have been proposed [19,23,47,48,52,74–88] including: i) dorsal and basal dural lymphatics ii) the olfactory bulb-cribriform lymphatic axis, iii) the choroid plexus, and iv) egress via spinal pathways [51,80,89]. (S3 Fig). Recent data demonstrate discontinuities in the arachnoid barrier at bridging veins, enabling drainage into the dura and subsequent exit via dural lymphatics [90]. Perivascular and meningeal macrophages may exit with CSF into meningeal lymphatics or along cranial nerves, ultimately reaching the dCLN [50,51,91]. Consistent with this, we detected SPION⁺ macrophages in the dCLN but fewer in the superficial cervical lymph nodes, which likely drain cranial nerve–associated routes [50,92]. Choroid plexus macrophages interface with both blood and CSF; stromal macrophages can exit via perivenous routes or migrate through the choroid plexus into the CSF and subsequently into the subarachnoid space. Kolmer macrophages likewise may exit via CSF drainage pathways [19,48,49,79]. Immune cells may also leave the CNS via spinal CSF pathways, including arachnoid villi draining into spinal veins [93–95], along spinal nerve roots to epidural lymphatics [96,97], and routes through the arachnoid layer to the spinal meninges to dural lymphatics [98,99]. Supporting this, we observed SPION⁺ macrophages in lumbar lymph nodes, not only in cervical nodes. (S1 Fig). The higher abundance of SPION⁺ macrophages in the dCLN relative to superficial cervical lymph nodes suggests dynamic CNS drainage through deep cervical pathways. Overall, the dCLN is the most dynamic with regard to CNS macrophage turnover likely because it is closest to the CNS, more so than other sites like other LN, and the spleen. Overall, macrophage egress—including of potentially SIV-infected cells—may be critical for understanding CNS reservoir dynamics and the redistribution of viral material to the periphery.

### Persistence and rebound of SIV-infected macrophages during ART and following ART interruption

An important question in this study is whether SIV-infected macrophages within the CNS can traffic to the periphery. In SIVE animals, we identified SIV-RNA⁺ SPION⁺ perivascular, meningeal, and choroid plexus macrophages, as well as SIV-RNA⁺ SPION⁺ macrophages in the dCLN, spleen, and DRG. Although the absolute numbers were low—likely due to the limited tissue volumes analyzed and low animal numbers— their presence supports the concept that virally infected CNS macrophages can exit to peripheral tissues. Future studies using whole-tissue analyses and magnetic enrichment of SPION-containing cells will likely strengthen this conclusion.

With ART, SIV-RNA⁺ SPION⁺ macrophages were eliminated from CNS parenchymal (perivascular) and peripheral tissues, but scattered SIV-RNA⁺ and gp41 ⁺ macrophages persisted in the meninges and choroid plexus. Following ART interruption, SIV-RNA⁺ SPION⁺ macrophages rebounded in the meninges, choroid plexus, spleen, and in plasma, but not within the CNS perivascular compartment. In our short ART regimen with CD8 depletion, plasma viremia did not fully suppress to undetectable levels, although most tissue-associated SIV RNA and DNA were cleared. At necropsy, FAC-sorted monocytes from ART animals lacked detectable SIV RNA by sensitive PCR assays. Regardless, not clearing SIV-RNA likely is a reason we have a rebound in the CNS meninges four weeks post ART interruption, although we did not see a rebound in the CNS perivascular macrophages in this same time window.

We did not identify SIV-DNA⁺ SPION⁺ macrophages within or outside the CNS under ART. This was unexpected, but most likely reflects the lower sensitivity of DNAscope relative to RNAscope in our hands. Using PCR on FAC-sorted monocytes, we consistently detected lower SIV DNA levels than SIV RNA, consistent with prior observations. Notably, Clements and colleagues recently demonstrated that virologically suppressed individuals harbor persistent, replication-competent HIV in monocyte-derived macrophages in blood [11]. Their findings parallel ours in that viral DNA levels may be low, yet macrophages retain replication-competent virus despite ART [11]. With ART interruption, we observed a rebound of virally infected macrophages in CNS meninges, spleen, and plasma, but not in perivascular macrophages nor in PBMC-associated SIV RNA or DNA. This pattern may reflect differential recruitment or replacement of macrophages in the meninges and choroid plexus during rebound. With longer ART durations, it is possible that perivascular macrophages would also exhibit viral rebound.

Taken together, these data suggest that during AIDS and SIVE, ongoing reseeding of the periphery from the CNS viral reservoir may occur. ART eliminates this reseeding, while ART interruption allows viral rebound from plasma, meninges, and spleen. Historically, the CNS has been considered an immune-privileged site with limited immune cell trafficking. However, macrophage egress plays a critical role in regulating inflammation in other tissues, and our findings indicate similar processes occur in the CNS during viral infection. Macrophages dynamically migrate from the bone marrow to the CNS, accumulate during inflammation, and can subsequently traffic out, carrying viral antigens or virus. The egress of macrophages with ART likely contributes to the resolution of inflammation and reduced macrophage infection. Although monocyte/macrophage trafficking has long been proposed, the present work provides the first demonstration of this phenomenon in a non-injury primate model of viral infection. Interestingly, preliminary laser capture microscopy data show SPION⁺ SIV-RNA⁺ macrophages in the bone marrow with viral sequences similar to those found in the brain (manuscript in preparation). Thus, macrophage egress from the CNS challenges conventional notions of immune privilege and likely contributes to neuroinflammatory regulation and CNS–periphery viral redistribution.

### Timing of CNS macrophage viral reservoir establishment

Using two fluorescently distinct SPIONs injected early (12–14 days post-infection) and late (30 days before necropsy), together with SIV-RNAscope, we examined when CNS macrophage viral reservoirs are established. AIDS and SIVE animals had significantly more early SPION⁺ SIV-RNA⁺ perivascular macrophages than late or dual-labeled cells. These early-labeled infected macrophages were eliminated with ART and did not rebound with ART interruption, consistent with our prior findings that the perivascular macrophage viral reservoir is established early in infection. In earlier work using dextran dyes to label CNS macrophages at early and late time points, we found that perivascular macrophages were labeled early—before lesion formation—but also acquired late dye and were found within SIVE lesions. This suggests that perivascular macrophages can migrate into lesions and may explain why SPION-labeled perivascular macrophages in our study were predominantly early-labeled.

We have previously used Tysabri (anti-VLA-4 α4β1 antibody) in animals with SIVE and MRI-visible CNS lesions; inhibition of leukocyte trafficking led to resolution of CNS lesions [36]. Whether Tysabri facilitates macrophage egress during lesion resolution remains unknown. In the meninges, we observed equal distributions of early-, late-, and dual-labeled SIV-RNA⁺ SPION⁺ macrophages in AIDS and SIVE animals. These were eliminated with ART and rebounded following ART interruption, consistent with ongoing recruitment of infected macrophages during disease and suppression of this recruitment with ART.

In the choroid plexus, late SPION⁺ SIV-RNA⁺ macrophages predominated in SIVE animals and were not eliminated by ART. This supports prior observations that monocyte trafficking through the choroid plexus is high and increases with SIV infection [33,38]. These data support the early establishment of the CNS macrophage viral reservoir, ART resolving CNS inflammation by inducing traffic out early, differential rates of viral seeding in the parenchyma, meninges, and choroid plexus, and increased traffic of virally infected SPION^+^ macrophages out to the periphery with AIDS. In CD8-depleted macaques, ART does not fully suppress plasma viral load to undetectable levels (∼10⁴ copies/mL). Viral RNA–positive monocytes may therefore persist in blood, potentially containing SPION, although we did not examine their SPION content. However, at necropsy, FAC-sorted monocytes from ART-treated animals lacked detectable SIV RNA and DNA by sensitive PCR. Overall, these data support: early establishment of the CNS macrophage viral reservoir; ART-induced resolution of CNS inflammation via enhanced macrophage egress; differential viral seeding rates in perivascular, meningeal, and choroid plus macrophage pools; and increased egress of viral infected macrophages to the periphery in AIDS.

### Choroid plexus: a putative gateway and reservoir for virus-infected macrophages

The choroid plexus is a key neuroimmune interface and a potential gateway for immune cell entry into the CNS [22,26,27,36,100]. HIV and SIV-infected cells are consistently observed in the choroid plexus, although its role as a viral reservoir remains uncertain. [26,27,101]. We observed accumulation of SPION⁺ macrophages in the choroid plexus, potentially due to diffusion of SPION from CSF across the epiplexus, recirculation of SPION, or infiltration of SPION⁺ monocytes/macrophages. [43]. Previously, we showed that blocking monocyte/macrophage trafficking with anti-VLA-4 α4β1 (Tysabri) prevented CNS viral reservoir establishment and reduced neuronal injury. Because VLA-4 is expressed on both vascular and CSF-facing sides of the choroid plexus, anti-VLA-4 may inhibit cellular trafficking between blood, CSF, and CNS parenchyma.

## Conclusion

Comparisons between the monkey model and human HIV-associated neurocognitive disorders (HAND) require caution. The SIV/CD8-depleted model used here has compressed disease kinetics with early inflammation, rapid lesion formation, and no behavioral correlate of HAND. Nonetheless, the model offers controlled timing of infection, the ability to label macrophage populations, and access to tissues at biopsy and necropsy. Our findings highlight that macrophage accumulation increases with infection, decreases with ART, and rebounds with ART interruption. The study provides direct evidence of macrophage trafficking into and out of the CNS during viral infection, helping to define the dynamics of CNS macrophage retention and egress in relation to inflammation. Overall, our data suggest that myeloid trafficking between the CNS and periphery plays a major role in both establishing and resolving CNS inflammation during HIV and SIV infection. Animals with SIVE had more SPION⁺ macrophages in the CNS, while ART-treated animals exhibited fewer CNS macrophages but increased trafficking to the periphery that occurred with CNS inflammation resolution. The possibility that CNS-derived, virally infected macrophages contribute to peripheral viral rebound challenges traditional assumptions about CNS immune privilege and highlights the dynamic, migratory behavior of CNS macrophages during SIV and HIV infection.

## Methods

### Ethics statement

All animal work was approved by the Tulane National Primate Research Center Care and Animal Use Committee. The TNRPC protocol number is 3497 and the animal welfare assurance number is A4499-01.

### Animals and viral infection

A total of 20 adult rhesus macaques (*Macaca mulatta*) born and housed at the Tulane National Primate Research Center in strict adherence to the “Guide for the Care and Use of Laboratory Animals” were used in this study (Fig 1A and Table 1). CD8^+^ lymphocytes were depleted to achieve rapid AIDS (3–4 months) with >75% incidence of SIVE, as previously described [33,34,72,102]. CD8^+^ T lymphocyte depletion was monitored longitudinally by flow cytometry and all macaques were persistently depleted (>28 days). Animals were experimentally infected intravenously (i.v) by inoculation with a bolus of SIVmac251 viral swarm (20 ng of SIV p28) provided by Dr. Ronald Desrosiers, over 5 min. At 21 d post-infection, n = 14 macaques began a 12–15-week regimen of antiretroviral therapy (ART) consisting of Raltegravir (Merck, 22 mg/kg) given orally twice daily, and Tenofovir (Gilead, 30 mg/kg) and Emtricitabine (Gilead, 10 mg/kg) combined in a sterile solution given once-daily, s.c. Ten animals were euthanized on ART and 4 were removed from ART for 4 weeks to allow for viral rebound. Of the n = 10 ART animals a subset of n = 4 were used for analysis of peripheral tissue analysis due to tissue availability. Animals were euthanized based on the recommendations of the American Veterinary Medical Association Guidelines for the Euthanasia of Animals upon developing signs of AIDS, which included: a > 15% decrease in body weight in 2 weeks or >30% decrease in body weight in 2 months; documented opportunistic infection; persistent anorexia>3 days without explicable cause; severe, intractable diarrhea; progressive neurological symptoms; or significant cardiac or pulmonary symptoms. SIV encephalitis (SIVE) was defined by the presence of multinucleated giant cells (MNGC) and the accumulation of macrophages [72]. Longitudinal plasma viral load (PVL) was assessed as previously described [103–105] to monitor viral suppression during treatment and rebound following ART interruption (Fig 1B). For PVL, 500μL of EDTA plasma was collected and plasma virions were pelleted by centrifugation (20,000 x g for 1 h). The sensitivity threshold of the assay was 100 copy Eq/mL with an average intra-assay coefficient variation of less than 25%. Log-transformed PVLs below the limit of detection were set to 0 for statistical analysis.

### SPION (and Dextrans)

Superparamagnetic iron oxide nanoparticles (SPION) were obtained from Bang Laboratories Inc. (PS-COOH Mag/Encapsulated, MEDG001, and MEFR001, Fishers, IN). SPION have internal fluorescence (Dragon Green [480/520] and Flash Red [660/690]) and are iron oxide nanospheres encapsulated in an inert polymer with an average particle size of 0.86μm diameter. SPION were prepared in a class 2 biosafety cabinet. 3 mL of stock solution and 7 mL of sterile, low endotoxin 1XPBS were added to a 15 mL conical tube and gently mixed. A magnet was used to separate the SPION from the liquid and an additional 7 mL of 1XPBS was added for a second wash. This process was repeated 7–10 X to ensure all original buffer was removed and the SPION were resuspended in a sterile 1XPBS solution. SPION reconstituted in 1mL of sterile 1XPBS, at a final concentration of 33 mg/mL. In animals receiving fluorescent Dextran (RITC-Dextran,10 KD, Invitrogen, CA) was administered at a concentration of 33 mg/mL.

### Intracisternal inoculation procedure

To avoid an increase in intracranial pressure, 1 mL of cerebrospinal fluid (CSF) was removed over a 5 min time period, from the cisterna magna prior to the inoculation of SPION or RITC-Dextran. Dragon Green [480/520] SPION (33 mg/mL) were administered 2 weeks post-inoculation at 12 or 14 dpi. Flash Red [660/690] SPION (33 mg/mL) (pseudo-colored blue) were injected late during infection prior to euthanasia (Range 3–22 days). SPION were administered in a single bolus over a 10minute time period to avoid undue CNS pressure and damage to ventricles and vasculature. This is a routine procedure in the veterinary services and follow up histopathology examination shows no signs of CNS tissue damage.

### Tissue collection and processing

Animals were anesthetized with ketamine-HCl and euthanized with i.v. pentobarbital overdose and exsanguinated. Blood was collected and Heparin Sulfate was administered i.v. and given 5 min to diffuse. Sodium pentobarbitol was administered via intracardiac stick and CSF was collected. Following CSF collection, animals underwent perfusion with 3L of chilled 1XPBS. Postmortem examination was performed by a veterinary pathologist that confirmed the presence of AIDS-defining lesions as previously described [33,34,72,102]. Brain (frontal, temporal, occipital, parietal, and choroid plexus) and peripheral tissues (cervical lymph nodes, spleen, and DRG) were: i) collected in zinc-buffered formalin and embedded in paraffin, ii) fixed with 2% paraformaldehyde for 4–48 hours, sucrose protected and embedded in optimal cutting temperature (OCT) compound for SPION analysis, or iii) snap frozen in OCT without fixation (spleen).

### Immunohistochemistry and immunofluorescence

IHC was performed as previously described using the antibodies targeting CD163^+^ (1:250, Leica (Deer Park, IL)), CD68^+^ (1:100, DAKO (Carpinteria, CA)), and IBA1^+^ macrophages (1:100, Wako (Osaka, Japan)) [33,34,102]. Briefly, formalin-fixed paraffin-embedded sections were deparaffinized and rehydrated followed by antigen retrieval with a citrate-based Antigen Unmasking Solution (Vector Laboratories, Burlingame, CA) in a microwave (900 W) for 20 min. After cooling for 20 min, sections were washed with Tris-buffered saline (TBS) containing 0.05% Tween-20 for 5 min before incubation with peroxidase block (DAKO, Carpinteria, CA) followed by protein block (DAKO, Carpinteria, CA) for 30 min and incubation with primary antibody. Following incubation with a peroxidase-conjugated polymer, slides were developed using a diaminobenzidine chromogen (DAKO, Carpinteria, CA) with Harris Hematoxylin (StatLab, McKinney, TX).

Immunofluorescence for CD163^+^, CD68^+^, IBA1^+^, and gp41^+^ macrophages was performed using antibodies and fluorochromes using CD163 (NCL-CD163 CE, AF568, Invitrogen (Carlsbad, CA)), CD68 (DAKO, AF568 (Carpinteria, CA)), IBA1 macrophages (Wako, DISCOVERY OmniMap Anti-Rb HRP (Osaka, Japan)), and gp41 (KK41, 1:100, NIH (Manassas, VA)) on 2% paraformaldehyde (PFA) fixed frozen sections. 2% PFA, fixed frozen sections were thawed for 20 min at room temperature, unwrapped, submerged in a citrate-based Antigen Unmasking Solution, and microwaved for one minute and forty-five seconds and cooled to room temperature. Slides were permeabilized in a solution of phosphate-buffered saline with 0.01% Triton X-100 and 0.02% fish skin gelatin (PBS-FSG-TX100) followed by a PBS-FSG wash, transferred to a humidified chamber and blocked with 10% normal goat serum (NGS) diluted in PBS-FSG for 40 min, followed by a 60-min primary antibody incubation, washes, and 40-minute secondary antibody incubation. Routine washes were performed and DAPI nuclear stain added for 10 minutes. Slides were mounted using a custom-formulated anti-quenching mounting media containing Mowiol (#475904, Calbiochem; San Diego, CA) and DABCO (#D2522, Sigma: St. Louis, MO) and allowed to dry overnight before being digitally imaged with a Zeiss Axio Scan.Z1. HALO software (HALO v3.4, Indica Labs; Albuquerque, NM) was used for quantification and analysis.

### Tissue viral RNA and DNA detection

Ultrasensitive SIV-RNAscope with probes for SIVmac251 was used to detect SPION-containing SIV-RNA^+^ cells within and outside of the CNS as previously described [102]. Tissue sections were placed in a target antigen retrieval solution, heated, and treated with protease plus, and a hydrogen peroxide blocker according to the manufacturer’s protocol (Advanced Cell Diagnostics, Newark, CA). SIVmac239 RNAscope probes (Advanced Cell Diagnostics, Newark, CA) were hybridized at 40°C in the HybEZ II Hybridization System. The RNAscope 2.5 HD Assay amplification steps were applied according to the manufacturer’s protocol. Target RNA was visualized through the addition of chromogenic Fast Red A and Fast Red B (Advanced Cell Diagnostics, Newark, CA), and sections were counterstained with hematoxylin (Sigma-Aldrich) and mounted using Vectamount (Vector Laboratories). Viral RNA-processed sections were subsequently stained for CD163^+^ or CD68^+^ macrophages using primary antibodies and IHC methods described above. Prussian blue iron staining and/or the internal fluorescence of SPION was used to detect vRNA^+^SPION-containing CD163^+^ macrophage. DNAscope was performed, as previously described [102,106,107]. SIV-DNA was detected in situ using an SIV-DNA sense probe (Advanced Cell Diagnostics, Newark, CA) for RNAscope Assay on 3 – 4 CNS cortical sections and 1 peripheral tissue (dCLN, spleen, and DRG) per animal. To reduce non-specific signal, brain tissues were pre-treated with 2N HCL for 30 min at room temperature.

### Detection and quantification of SPION-containing macrophages in tissues

SPION were detected in the central nervous system (CNS) and peripheral tissues by 1) light microscopy by morphology of the amber SPION beads, 2) Prussian blue iron staining (Sigma Aldrich Iron Stain, St. Louis, MO), and 3) internal fluorescence of Dragon Green or Flash Red (artificially colored blue). The number of SPION-containing macrophages with IHC for macrophages with CD163^+^ or CD68^+^ was counted. SPION+ macrophages in whole tissue sections were counted manually at 20x by light microscopy (Plan-Apochromat 620/0.7, Olympus; Japan) in a blinded fashion. Whole section tiling and stitching was done using a Zeiss Axio Imager M1 microscope (Zeiss; Oberkochen, Germany) with AxioVision (Version 4.8, Zeiss; Oberkochen, Germany) using Plan-Apochromat 620/0.8 and 640/0.95 Korr objectives followed by manual annotation of the parenchyma from the meninges into separate regions of interest (ROI) and tissue area reported as mm^2^.

For analysis of early versus late SPION^+^ macrophages and virally infected SPION^+^ macrophages, whole slide fluorescent images of stained sections were scanned using a Zeiss AxioScan Z.1 (Carl Zeiss MicroImaging, Inc., Thornwood, NY). Scanned sections were analyzed with HALO modular analysis software (HALO v3.4, Indica Labs; Albuquerque, NM). The parenchyma and meninges were first annotated into separate ROI/annotation layers and the number of early, late, and dual SPION-containing macrophages and vRNA-FISH early, late, and dual SPION-containing macrophages were counted using the FISH v.3.2.3 module (HALO v3.4, Indica Labs; Albuquerque, NM) and reported as the number of cells per mm^2^.

### Cell associated viral load

A minimum of 100,000 T cells and Monocytes were isolated from frozen PBMCs using flow cytometry using a BD Aria (S2 Fig) as previously described [56,57]. Thawed PBMCs were incubated with fluorochrome-conjugated antibodies to isolate T cells and Monocytes including anti-C14-FITC (clone: HCD14, BioLegend), anti-CD3-PE (clone: UCHT1, BioLegend), anti-HLA-DR-ECD (clone: Immu-357, Beckman), anti-7-AAD-PECY5 (BD), anti-CD20-PERCPCY5.5 (clone: 2H7, BD Pharmingen), and anti-CD3-PECY7 (clone: SK7, BioLegend). Monocytes were selected based on size and granularity (FSC vs SSC) followed by selection of live (7-AAD^+^) CD14^+^HLA-DR^+^CD3^-^ CD20^-^ cells and T cells were likewise selected based on size and granularity (FSC vs SSC) followed by selection of live (7-AAD^+^) CD3^+^ HLA-DR^-^CD14^-^ cells (S2 Fig). After sorting, cells were washed with 2% FBS-PBS and dry pelleted before cell-associated SIV viral load analysis was performed by The Quantitative Molecular Diagnostics Core at the Fredericks National Laboratory. Briefly, the Quantitative Molecular Diagnostics Core performed total DNA and RNA extraction from the sorted Monocytes and T cells and tested them separately using a hybrid assay format combining quantitative real-time standard curve interpolation and Poisson-based methods. Extracted RNA was resuspended and divided among 10 replicate quantitative polymerase chain reaction (qPCR) or quantitative reverse transcription polymerase chain reaction (qRT-PCR) reactions. SIV DNA testing was multiplexed with an assay for a single-copy genomic sequence from rhesus macaque CCR5 to normalize to cell numbers based on diploid genome equivalents. For specimens where all wells yielded positive PCR reactions, the SIV DNA copy number was calculated using the average of the standard curve interpolated values and normalized to cell numbers. When not all wells were positive, Poisson methods were used to calculate viral copy numbers, followed by normalization. SIV RNA values were determined by qRT-PCR and normalized to cell numbers based on CCR5 DNA copy numbers, accounting for near-quantitative RNA and DNA recovery with the employed extraction methods.

### Statistical analysis

Statistical analyses were performed using Prism version 10.0 (GraphPad Software; San Diego, CA). Comparisons between animals with SIVE, ART, and following ART interruption were made using a nonparametric one-way analysis of variance (Kruskal-Wallis, GraphPad Software; San Diego, CA) with Dunn’s multiple comparisons. Statistical significance was accepted at p < 0.05 and all graphing was done using Prism (GraphPad Software; San Diego, CA).

## Supporting information

S1 Fig Lumbar lymph node. Macrophages containing brown granular pigment (SPION) are present within medullary cords and sinuses (arrows). Bar = 50 μm. H&E. Data presented here are representative of n = 4 animals.
(TIFF)

S2 Fig Purity of sorted CD3 + T Cells and CD14 + monocytes for cell-associated viral load analysis. Total lymphocytes and monocytes are gated initially based on FSC versus SSC (A), and doublets are excluded (B), followed by exclusion of dead cells and CD20 + B lymphocytes by negative selection (C). CD3 + T lymphocytes and monocytes are initially selected using CD3 and HLA-DR (D), followed by CD14 and CD16 for monocytes (HLA-DR + , CD3-) (E). Post-purity analytical analysis is performed after cell sorting and yields 98.9% purity for CD3 + T cells (F) and 98.1% for monocytes (G) based on ungated data. Results presented here are from one animal and are representative of n = 14. Numbers are percentages of each population within the same dot plot.
(TIFF)

S3 Fig Schematic illustration of potential pathways for macrophages to traffic out of the CNS. Macrophages and other immune cells may exit the CNS via several distinct anatomical routes: (i) Dorsal and basal dural lymphatics, which drain cerebrospinal fluid (CSF) and associated immune cells to peripheral lymph nodes; (ii) Olfactory bulb–cribriform plate axis, facilitating migration through the nasal lymphatics into cervical lymph nodes; (iii) Choroid plexus, which acts as a gateway between the blood, CSF, and immune compartments; (iv) Spinal pathways, allowing drainage along spinal nerve roots and into peripheral lymphatics. These routes ultimately lead to regional lymph nodes such as the deep cervical lymph nodes (a), superficial cervical lymph nodes (b), and lumbar lymph nodes (c), where antigen presentation, viral infection, and immune surveillance can occur. This figure was created using BioRender.
(TIFF)

S1 Table Cell count data for Fig 2. (XLSX)

S2 Table Cell count data for Fig 5. (XLSX)
