# Supplementary figures and images for: Resolving inflammation: The impact of antiretroviral therapy on macrophage traffic in and out of the CNS
Source: PLoS Pathog. 2025 Dec 1;21(12):e1013180. doi: 10.1371/journal.ppat.1013180 (PMC12677798; doi:10.1371/journal.ppat.1013180)

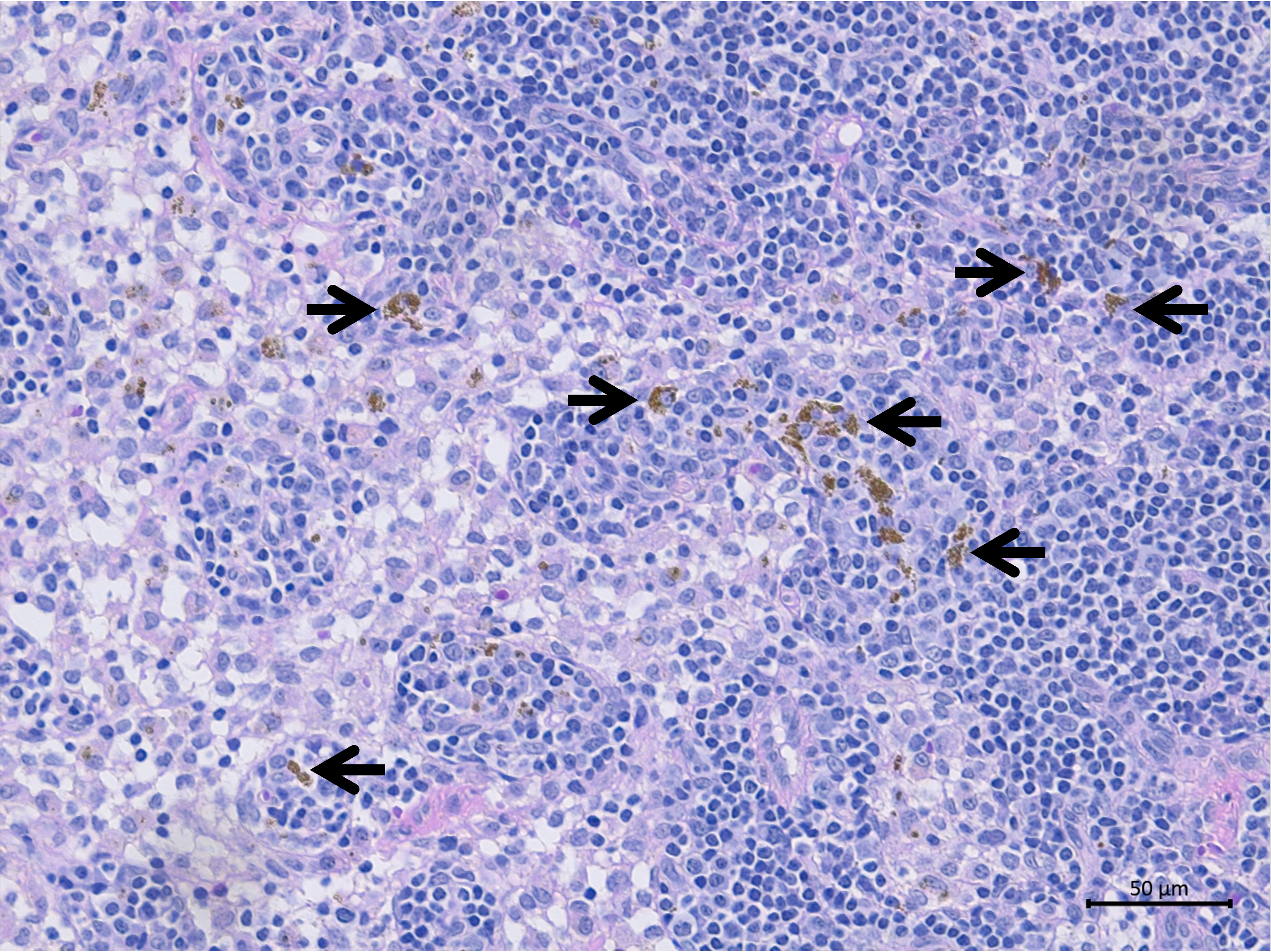

Supplement: S1 Fig — Macrophages containing brown granular pigment (SPION) are present within medullary cords and sinuses (arrows). Bar = 50 μm. H&E. Data presented here are representative of n = 4 animals. (TIFF) [file ppat.1013180.s001.tiff]

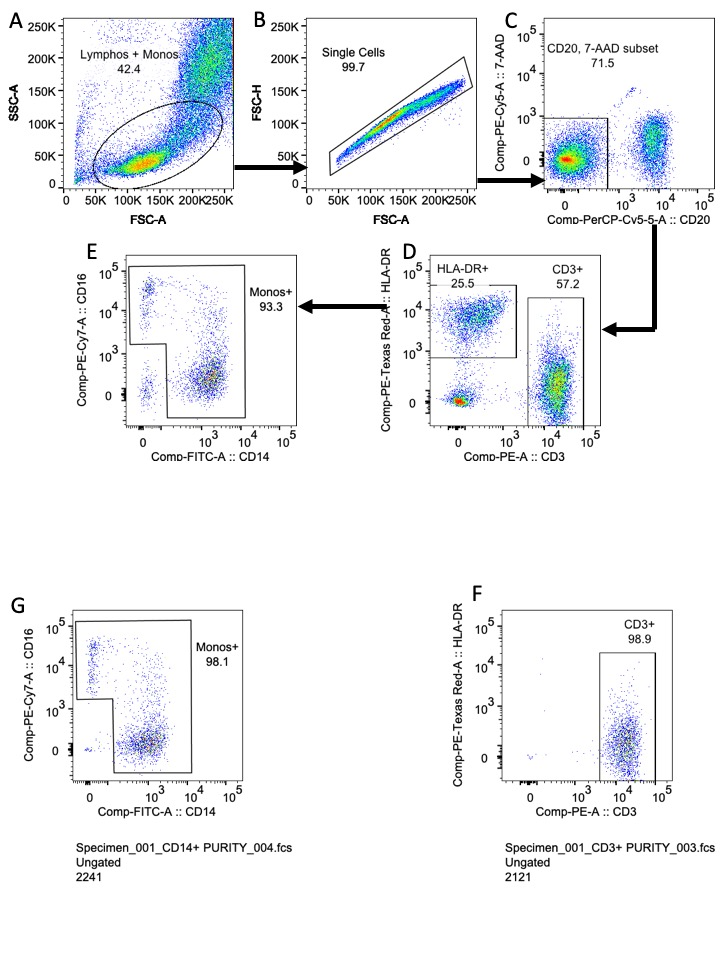

Supplement: S2 Fig — Total lymphocytes and monocytes are gated initially based on FSC versus SSC (A), and doublets are excluded (B), followed by exclusion of dead cells and CD20 + B lymphocytes by negative selection (C). CD3 + T lymphocytes and monocytes are initially selected using CD3 and HLA-DR (D), followed by CD14 and CD16 for monocytes (HLA-DR + , CD3-) (E). Post-purity analytical analysis is performed after cell sorting and yields 98.9% purity for CD3 + T cells (F) and 98.1% for monocytes (G) based on ungated data. Results presented here are from one animal and are representative of n = 14. Numbers are percentages of each population within the same dot plot. (TIFF) [file ppat.1013180.s002.tiff]

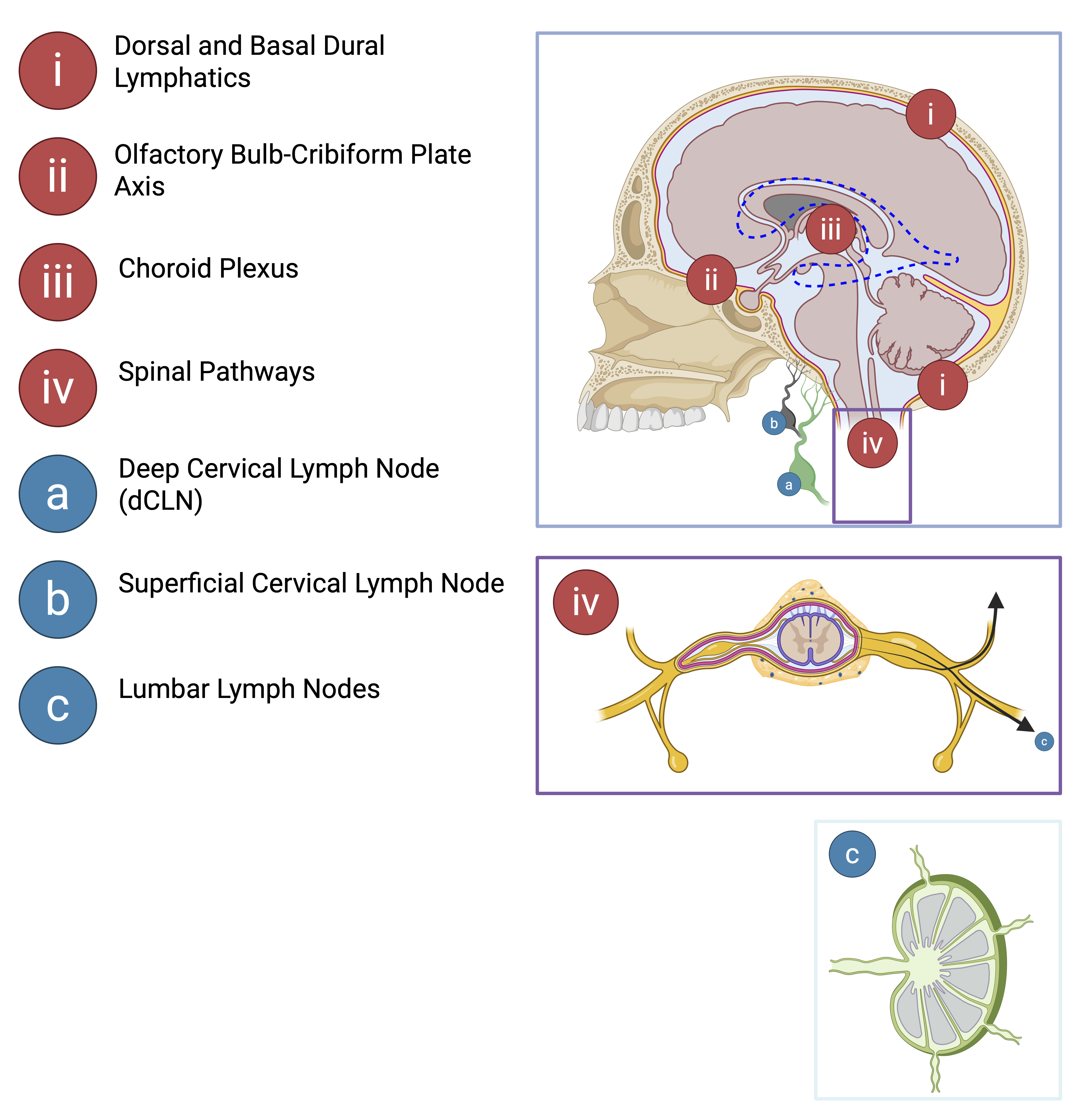

Supplement: S3 Fig — Macrophages and other immune cells may exit the CNS via several distinct anatomical routes: (i) Dorsal and basal dural lymphatics, which drain cerebrospinal fluid (CSF) and associated immune cells to peripheral lymph nodes; (ii) Olfactory bulb–cribriform plate axis, facilitating migration through the nasal lymphatics into cervical lymph nodes; (iii) Choroid plexus, which acts as a gateway between the blood, CSF, and immune compartments; (iv) Spinal pathways, allowing drainage along spinal nerve roots and into peripheral lymphatics. These routes ultimately lead to regional lymph nodes such as the deep cervical lymph nodes (a), superficial cervical lymph nodes (b), and lumbar lymph nodes (c), where antigen presentation, viral infection, and immune surveillance can occur. This figure was created using BioRender. (TIFF) [file ppat.1013180.s003.tiff]
